# Keeping Meta-Analyses Hygienic During the COVID-19 Pandemic

**DOI:** 10.3389/fpubh.2021.722458

**Published:** 2021-09-29

**Authors:** Jordane Boudesseul, Oulmann Zerhouni, Allie Harbert, Clio Rubinos

**Affiliations:** ^1^Facultad de Psicología, Instituto de Investigación Científica, Universidad de Lima, Lima, Peru; ^2^University Paris Nanterre, Nanterre, France; ^3^School of Medicine, University of North Carolina at Chapel Hill, Chapel Hill, NC, United States; ^4^Department of Neurology, University of North Carolina at Chapel Hill, Chapel Hill, NC, United States

**Keywords:** COVID−19, meta-analysis, heterogeneity, publication bias, hydroxychloroquine

## Abstract

Despite the massive distribution of different vaccines globally, the current pandemic has revealed the crucial need for an efficient treatment against COVID-19. Meta-analyses have historically been extremely useful to determine treatment efficacy but recent debates about the use of hydroxychloroquine for COVID-19 patients resulted in contradictory meta-analytical results. Different factors during the COVID-19 pandemic have impacted key features of conducting a good meta-analysis. Some meta-analyses did not evaluate or treat substantial heterogeneity (*I*^2^ > 75%); others did not include additional analysis for publication bias; none checked for evidence of *p*–hacking in the primary studies nor used recent methods (i.e., *p*-curve or *p*-uniform) to estimate the average population-size effect. These inconsistencies may contribute to contradictory results in the research evaluating COVID-19 treatments. A prominent example of this is the use of hydroxychloroquine, where some studies reported a large positive effect, whereas others indicated no significant effect or even increased mortality when hydroxychloroquine was used with the antibiotic azithromycin. In this paper, we first recall the benefits and fundamental steps of good quality meta-analysis. Then, we examine various meta-analyses on hydroxychloroquine treatments for COVID-19 patients that led to contradictory results and causes for this discrepancy. We then highlight recent tools that contribute to evaluate publication bias and p-hacking (i.e., *p*-curve, *p*-uniform) and conclude by making technical recommendations that meta-analyses should follow even during extreme global events such as a pandemic.

## Background

The 2020 COVID-19 pandemic has highlighted the urgent need for the development and administration of a new treatment for COVID-19. Despite the rollout of several different vaccines globally, the need to find treatment remains essential given the uncertainty and shortcomings with equal distribution of vaccines and vaccine availability. Meta-analyses have historically been used to establish the existence, size, and confidence of therapeutic effects or causes of particular diseases.

Meta-analysis is an important tool to determine the effectiveness of COVID-19 treatments, but it is essential that the strength of evidence be maintained by adhering to all components of the methodology. Various factors during the COVID-19 pandemic, including time pressure, have resulted in alterations and omissions of key aspects of meta-analysis that lower the quality of evidence. Some meta-analyses did not evaluate publication bias, nor treat substantial heterogeneity (*I*^2^ > 75%); none checked for evidence of *p*–hacking in the primary studies nor used recent techniques (i.e., *p*-curve or *p*-uniform) to estimate average population-size effect. Journals greatly favor publishing significant findings in comparison to non-significant findings, resulting in publication bias which can overestimate effect sizes (the strength of the relationship between two variables). These discrepancies may contribute to opposing results in the research evaluating COVID-19 treatments. A prominent example of this is the use of hydroxychloroquine (HCQ), where some studies reported a large protective effect, whereas others indicated no significant effect or even increased mortality when HCQ was administered with the antibiotic azithromycin.

In this paper, we first highlight the benefits and fundamental steps of meta-analytical studies. Then, we analyze examples of meta-analyses of HCQ treatments for COVID-19 patients that led to contradictory results and causes for this discrepancy. We conclude by making recommendations that meta-analyses should follow even during extreme global events such as a pandemic (see Table 1).

**Table 1:** General recommendations for meta-analysis of clinical studies.

1. Include published and unpublished studies on the basis of inclusion/exclusion criteria (e.g., designs, measures, sample characteristics). Ideally, pre-register your meta-analysis on an accessible server (1) (e.g., PROSPERO database, Open Science Framework)
2. Systematically run heterogeneity tests (*Q* statistic, the variance between studies (τ^2^), and the relationship between the real heterogeneity and the total variation observed, *I*^2^). Some depend on the number of participants (*Q*) whereas other depends on the metric scale (τ^2^) so it is crucial to compare them to estimate true heterogeneity (2, 3)
3. In case of substantial heterogeneity (i.e., *I*^2^ > 75%), create homogenous subgroups based on theoretical or methodological justifications (4)
4. Estimate publication bias using funnel plots and inferential tests (i.e., Begg's/Egger's tests). In case of publication bias, run additional analysis comparing the main results with/without these studies (2, 3)
5. Evaluate *p*-hacking using *p*-curve. If H0 is true (no effect), the *p*-distribution must be uniform but right-skewed if there is an effect. In case of signs of *p*-hacking, exclude those studies and run again the analysis to compare the results (5)
6. Conduct separate analyses for observational, quasi-experimental, and experimental studies and evaluate the risk of bias for each study (6).

## Methods

### Meta-Analysis: Principles and Procedures

Meta-analysis involves a set of statistical techniques to synthesize effect sizes of several studies on the same phenomenon (2, 3). Several benefits are expected from clinical meta-analyses:

- Identify, screen, select, and include studies based on systematic reviews of the literature (i.e., as recommend by the PRISMA Statement) (7).
- Compute the mean effect sizes across different studies (i.e., the average effect of a particular treatment on a specific condition).
- Evaluate the level of heterogeneity (i.e., the amount of variation in the outcomes detected by the different studies).
- Determine the impact of publication bias (i.e., the lack of publication of negative trials and underrepresentation of unpublished data, can lead to overestimated effect sizes).
- Run meta-regressions and subgroup analyses to control for the effects of studies' characteristics (e.g., design, procedure, measures) and sample (e.g., age/gender, BMI, clinical history).

Meta-analyses begins by identifying, screening, and evaluating potentially relevant studies, and ultimately collecting data from included studies and evaluating their quality (through PRISMA, for instance) (7). The mean and variance of the estimates is collected from every included study to compute a global weighted mean based on the inverse variance (2, 3). Some recent meta-analyses on the effect of HCQ in COVID-19 patients have omitted basic practices to assess publication bias (8), resulting in massive untreated heterogeneity (8) (i.e., *I*^2^ > 80%) or meta-analyzed small sets of studies [*k* ≤ 3 implying low statistical power (9)]. None used recent tools to evaluate *p*-hacking and recent techniques for assessing the publication bias, possibly leading to an overall biased representation of the population-size effect.

### Gathering the Studies

The first step in conducting a meta-analysis is to search the literature for studies investigating a specific predefined question and using predetermined inclusion and exclusion criteria based on theoretical or methodological criteria (7) to determine eligibility. Several methods exist to assess and correct for publication bias among a set of studies (e.g., Egger or PET-PEESE tests, *p*-uniform, *p*-curve) (5, 10). A general issue is that studies likely differ significantly in design (e.g., randomized controlled trial vs. observational studies) and the specific questions they investigate (e.g., viral load, shedding, mortality/ICU events, mild symptoms). For instance, investigators must decide whether observational or quasi-experimental studies should be included alongside experimental studies. The lack of randomization inherent to observational or quasi-experimental studies is problematic as they are at risk of bias by uncontrolled confounding variables (11).

Recent advances in techniques question the reliance on estimates presented in the original studies for meta-analysis due to significant limitations (12). Systematic reviews and meta-analyses that use individual patient data (IPD) suggest collecting, validating, and reanalyzing the raw data from all clinical trials included in the meta-analysis. Following COCHRANE recommendations, IPD meta-analyses offer a multidisciplinary and cross-cultural perspective that decreases cultural and professional biases. IPD analyses also enable better assessment of moderator variable impact and improves statistical power (13). Integrating contextual variables helps ensure main effects are not explained by sample or country characteristics (or any other contextual factors). Limitations of including such variables are potential collinearity with other variables or insufficient precision in the measure. Although there are several advantages of conducting IPD meta-analyses, it also requires significant organization and coordination that can be challenging.

### Statistical Power

A major strength of meta-analysis is the relatively high statistical power associated with compiling several studies (i.e., independent RCTs may have few participants per group limiting their statistical power). The median number of studies in the Cochrane Database for Systematic Reviews is six, according to Borenstein et al. (2). This is a serious concern considering that (1) subgroup analysis and meta-regressions are routine procedures that require high levels of statistical power, and (2) many meta-analyses have high heterogeneity (*I*^2^ > 75%), which negatively affects precision and thus statistical power. For example, the statistical power to detect a small effect size (*d* = 0.2) with 25 participants per group in 6 different studies using a random-effects model with moderate heterogeneity (*I*^2^ = 50%) and a 5% of type I error is only 26.7% (https://bookdown.org/MathiasHarrer/Doing_Meta_Analysis_in_R/power-calculator-tool.html).

### Assessing Heterogeneity

The objective of meta-analysis is not simply to calculate an average weighted effect estimate but also to make sense of the pattern of effects. An intervention that consistently reduces the risk of mortality by 30% in numerous studies is different from an intervention that reduces the risk of mortality by 30% on average, with a risk reduction ranging from 10 to 80% across studies. We must determine the true variance to provide different perspectives on the dispersion of the results based on the *Q* statistic, the variance between studies (τ^2^), and the relationship between the real heterogeneity and the observed total variation (*I*^2^).

### Publication Bias

Publication bias affects both researchers conducting meta-analyses and physicians searching for primary studies in a database. If the missing studies are a random subset of all studies, excluding them will result in less evidence, wider confidence intervals, and lower statistical power, but will not have a systematic influence on the effect size (2). However, whenever there are systematic differences in unpublished and published studies included in the meta-analysis, the weighted effect sizes are biased (e.g., a lack of studies reporting non-significant effects of HCQ in COVID-19 patients). Dickersin (14) found that statistically significant results are more likely to be published than non-significant findings, and thus when published studies are combined together, they may lead to overestimated effects. Also, for any given sample size, the result is more likely to be statistically significant if the effect size is large. Studies with inflated estimate effects are expected to be reported in the literature more frequently as a result (i.e., the first studies on HCQ likely reported large effects). This trend has the potential to produce large biases both on effect size estimates and significance testing (15).

Different techniques have been developed to detect publication bias (16). A widely used method—the funnel plot—consists of plotting effect sizes against their standard errors or precisions (the inverse of standard errors). A skewed funnel plot is usually an indication of the presence of publication bias. However, subjective visual examination as well as coding of the outcome, the choice of the metric, and the choice of the weight on the vertical axis all impact the appearance of the plot (17). Inferential tests such as Egger's regression test regress the standardized effect size on the corresponding precisions (the inverse of the within-study variance). Although widely used, the Egger test may suffer from an inflated type I error rate or low statistical power in certain conditions (16, 17).

## Results

### Hydroxychloroquine and COVID-19 Meta-Analysis

HCQ and chloroquine (CQ) have been used for decades to manage and treat malaria and several autoimmune conditions. At the beginning of the pandemic, preliminary studies (18–20) suggested that HCQ might have a positive effect on the treatment of COVID-19 patients. This led the U*.S. Food and Drug Administration* (FDA) to issue an emergency-use (EUA) authorization on March 28, 2020 allowing for HCQ sulfate and CQ phosphate to be donated to the *Strategic National Stockpile* for use in hospitalized COVID-19 patients. Given its multiple antiviral effects, it is plausible that HCQ could be beneficial in COVID-19 patients (21). *In vitro* data have shown that HCQ/CQ blocks viral infection by inhibiting virus/cell fusion through increasing endosomal pH (22) and by reducing the production of inflammatory cytokines (23–25). Shortly after the EUA of HCQ/CQ, a group in France published a study describing viral load reduction/cure with HCQ (20). However, this study included a small sample size, was non-randomized, only reported viral load as an outcome, and excluded the most severely ill patients from the analysis. Numerous meta-analyses have already been published on the use of HCQ in COVID-19 patients, with some indicating a large protective effect for HCQ (8), and others reporting no effect (9) or increased mortality when HCQ was used with the antibiotic azithromycin (AZ) (26) (see a complete Table of HCQ/CQ meta-analysis and their bias on Table 2).

**Table 2:** Meta-analysis on the efficacy of hydroxychloroquine on COVID-19 patients published in peer-reviewed journals.

**References**	***k/N***	**Main results**	**Heterogeneity analysis**	**Publication bias analysis**
Ayele Mega et al. (27)	20/6,782	HCQ group did not differed on the rate of virologic cure (OR = 0.78; 95% CI [0.39–1.56]) or the risk of mortality (OR = 1.26; 95% CI [0.66–2.39]) compared to control.	Some analysis revealed high heterogeneity (up to *I*^2^ = 95%). Subgroup analysis of observational vs. RCTs studies.	Cochrane risk of bias tool for RCTs. Newcastle-Ottawa Quality Assessment scale (NOS) for observational studies. Subgroup analysis with low biased studies.
Bignardi et al. (28)	12/7,629	HCQ (with or without AZ) was not associated with mortality (RR = 1.09, [0.98–1.20]).	Moderate heterogeneity (*I*^2^ ≤ 54.6%). Subgroup analysis based on sensitivy analysis.	Egger test did not revealed sign of publication bias (*p* > 0.05).
Choudhuri et al. (29)	14/12,455	HCQ did not affect mortality compared to control group (RR = 1.003, [0.983–1.022]).	Low to high heterogeneity (up to *I*^2^ = 97.9%). No subgroup analysis.	Two authors independently evaluated within-study bias.
Das et al. (30)	7/726	HCQ did not affect the virological cure except after day 5 (OR = 9.33, [1.51–57.65]).	Null (*I*^2^ = 0%) to high heterogeneity (*I*^2^ = 96%) but small set of comparisons (*k* = 2).	Cochrane handbook to assess biased of RCTs (2 independent authors) and NOS for observational studies. ROBINS-I tool for non-randomised trials.
Ebina-Shibuya et al. (31)	8/2,063	HCQ was not associated with mortality (OR = 1.05, [0.53–2.09]).	*I*^2^ varies between 0 (adverse event), 31% (all cause death), 57% (time to viral clearance) up to 74% (for viral clearance at 7 days). Subgroup analysis on study design (RCTs vs. observational).	Cochrane Risk of Bias tool for RCTs.
Elavarasi et al. (32)	15/10,659	No significant reduction in mortality in HCQ group (RR = 0.98, [0.66–1.46]), fever duration (mean difference – 0.54 days) or clinical deterioration (RR = 0.90, [0.47–1.71]).	High heterogeneity for mortatliy and clinical deterioration (*I*^2^ = 87%), virological clearance (*I*^2^ = 80%), time to fever remission (*I*^2^ = 72%). Subgroup analysis of RCTs vs Cohort studies.	Cochrane Risk of Bias Tool for RCTs/Newcastle Ottawa Scale revealed significant bias without additional analysis.
Elsawah et al. (33)	6/609	No significant effect on viral clearance, clinical progressions, or mortality (*p's* > 0.10). Significant improvement on radiological progression (risk difference −0.20 [−0.36, −0.03]).	Low (*I*^2^ = 0%) to high heterogeneity *(I*^2^ = 94%) without subgroup analysis.	Cochrane Risk of Bias Tool (2 independent authors). Sensitivity analysis after removing the low-quality studies.
Kashour et al. (34)	21/20,979	No effect of HCQ on mortality (OR = 1.05, [0.96–1.15]) and small increased mortality with HCQ/AZ combination on a subset of studies (OR = 1.32, [1.00–1.75]).	No heterogeneity for the HCQ group and moderate for the HCQ/AZ comparison (*I*^2^ = 68.1%). Sensitivy analysis excluded studies with high risk of bias.	Neither funnel plot nor Egger's regression test revealed signs of publication bias (*p* = 0.276)
Fiolet et al. (26)	17/11,932	No difference in mortality for all studies or RCT (OR = 0.83 and 1.09).	*I*^2^ = 84% among non-RCTs with null heterogeneity for RCTs.	Funnel plot, Begg's and Egger's tests.
Ghazy et al. (35)	14/12,821	No difference between standard care en HCQ group (RR = 0.99, [0.61–1.59]). Mortality higher in HCQ/AZ comparison (RR = 1.8, [1.19–2.27]).	High heterogeneity was observed in different analysis (0% < *I*^2^ <98%). Subgroup analysis no revealed significant effect (e.g., mortality HCQ/AZ, RR = 2.23, [1.70–2.91]).	Publication bias assed by funnel plot.
Hussain et al. (36)	6/381	The risk of mortality in HCQ treated individuals is on average 2.5 times greater than in non-HCQ individuals (95% CI [1.07–6.03]). For moderate to mild symptoms, the rate of improvement was 1.2 higher compared to the control group (95% CI [0.77–1.89]).	These studies were perfectly homogeneous (*I*^2^/τ^2^ = 0).	Marginal asymmetry on funnel plot.
Hong et al. (37)	14/24,780	No effet of HCQ alone or in combination on mortality (OR = 0.95, [0.72–1.26]).	Substantial heterogeneity in all analysis (71% ≤ *I*^2^ ≤ 93%). Subgroup analysis of HCQ alone without effect (OR = 0.90, [0.60–1.34]).	Publication bias visible on funnel plot. Comparisons results with and without biased studies (no significant differences).
Lewis et al. (38)	4/4,921	HCQ group were not at fewer risks of developing COVID-19 (RR = 0.82, [0.65–1.04]), hospitalization (RR = 0.72, [0.34–1.50]) or mortality (RR = 3.26, [0.13–79.74]) compared to control but increased the risk of adverse events (RR = 2.76, [1.38–5.55]).	Statistical heterogeneity was assessed using the χ^2^ and *I*^2^ statistics (either 0 or 95%). Subgroup analysis based on (1) location contact with COVID-19, (2) dose of HCQ, and (3) pre- vs. post-exposure prophylaxis. No heterogeinty available for subgroups.	Funnel plot was not assessed giving the small number of studies.
Million et al. (8)	20/105,040	HCQ effective on cough, duration of fever clinical cure death and viral shedding (OR = 0.19, 0.11, 0.21, 0.32, and 0.43).	*Q*-test for the all set of studies (*Q* = 51.8, *p* < 0.001). *I*^2^ ≥ 75% and significant *Q*-test for subset of studies when k > 2 studies (except for deaths, *I*^2^ = 0%, *p* = 0.071). No subset analysis based on heterogeneity.	None.
Patel et al. (39)	6/2,908	No difference between HCQ and control group on mortality (OR = 1.25, [0.65, 2.38]). Higher mortality in HCQ/AZ group compared to control (OR = 2.34, [1.63–3.34]).	There was significant heterogeneity in mortality outcome (*I*^2^ = 80%) for HCQ. Subgroup analysis based on sensitivity analysis. For the HCQ/AZ groups, there was perfect homogeneity (*I*^2^ = 0%).	Funnel plot was asymmetrical. Subgroup analysis based on homogeneous studies.
Pathak et al. (40)	7/4,984	No difference in outcome with/without hydroxychloroquine (OR = 1.11, [0.72, 1.69]).	Moderate heterogeneity (32% ≤ *I*^2^ ≤ 44%). No subgroup analysis.	Funnel Plot and Egger regression asymmetry test (although not available in the paper).
Putman et al. (41)	45/6693	HCQ use was not significantly associated with mortality (HR = 1.41, [0.83, 2.42]).	Low heterogeneity (*I*^2^ = 0–32%) but small set of studies (*k* = 2 or 3).	Newcastle-Ottawa Scale for cohort studies and the Risk of Bias 2.0 tool for randomized controlled trials; case series assumed to be high risk by default.
Sarma et al. (9)	7/1,358	No differences on viral cure (OR = 2.37, [0.13–44.53]), death/clinical worsening (OR = 1.37, [1.37–21.97]) or safety (OR = 2.19, [0.59–8.18]).	Heterogeneity varies from null (for safety issues) to high (*I*^2^ = 72%, for virological cure). No subset analysis (except for the inclusion/exclusion of Gautret et al. [22]).	Cochrane/ROBINS-I/Newcastle Ottawa Scale (3 researchers).
Shamshirian et al. (42)	37/45,913	No difference on mortality in HCQ group (RR = 0.86, [0.71–1.03]) or HCQ/AZ comparison (RR = 1.28, [0.76–2.14]).	High heterogeneity (*I*^2^ = 87–90%). Meta-regressions indicated significant effect of age (*p* < 0.001).	Moderate publication bias for mortality based on Egger's test (*p* = 0.02).
Singh et al. (43)	7/746	No benefits of HCQ on viral clearance (RR, 1.05; 95% CI, 0.79 to 1.38; *p* = 0.74). Significantly more deaths in the HCQ group compared to the control group (RR, 2.17; 95% 1.32 to 3.57; *p* = 0.002).	Moderate heterogeneity in the clearance analysis (*I*^2^ = 61.7%, *p* = 0.07) and none in the death analysis (*I*^2^ = 0.0%, *p* = 0.43). No subset analysis based on heterogeneity.	Trim and fill adjustment, rank correlation, and Egger's tests.
Ullah et al. (44)	12/3,912	Higher mortality (OR = 2.23, [1.58–3.13]) and net adverse events (OR = 4.59, [1.73–12.20]) in HCQ group compared to control.	Moderate to high heterogeneity (*I*^2^ = 54–94%) without subgroup analysis.	Funnel plot revealed minimal publication bias.
Yang et al. (45)	9/4,112	HCQ-azithromycin combination increased mortality in COVID-19 patients (OR = 2.34;, [1.63–3.36]) though it was also associated with benefits on viral clearance in patients (OR = 27.18, [1.29–574.32]). HCQ-alone did not reveal significant changes in mortality rate, clinical progression, viral clearance, and cardiac QT prolongation.	Null to high heterogeneity (*I*^2^ = 84%). Subsequent subgroup analysis showed that HCQ treatment could recuded mortality and severe illness in severely infected COVID-19 patients (OR = 0.27, [0.13–0.58]).	Funnel plot analysis did not reveal obvious publication bias. Possible bias due to lack of demographic and clinical data.
Zang et al. (46)	7/851	No difference in illness duration between the HCQ group and the standard treatment group (RR = 0.66, [0.18–2.43]). Death was higher in HCQ group compared to standard (RR = 1.92, [1.26–2.93]).	Moderate heterogeneity was observed (41.2% ≤ *I*^2^ ≤ 72.1%) without subgroup analysis.	Cochrane Risk of Bias Tool for RCTs evaluated quality of studies (2 reviewers). Newcastle Ottawa Scale for observational studies and Egger test.

*To date, 24 meta-analyses were published on April 11th, 2021. This table only described peer-reviewed meta-analyses evaluating HCQ efficacy on COVID-19 patients. We reported the number of studies (k) and participants (N) after exclusion/inclusion criteria*.

### Some Examples of Questionable Research Practices in Meta-Analysis Suggesting No-Effect or Effects of HCQ

Million et al. (8) published a meta-analysis containing 20 studies involving 105,040 patients of which 19,270 had been on chloroquine derivatives and found a positive effect of the drug on mortality and symptoms associated with COVID-19. On the other hand, Fiolet et al. (26) did not find any effect of HCQ/AZ in a meta-analysis of 29 studies including 11,932 patients. In both meta-analyses, the authors found large heterogeneity among the included studies (*I*^2^ = 75% and *I*^2^ = 83%), which suggests the presence of confounders not being accounted for across studies, and neither study performed subgroup analysis to better explore the high heterogeneity. Study selection was problematic in both studies. For instance, Million and colleagues did not publish a flow diagram with the different phases of a systematic review as recommended by the PRISMA Statement (7). Several items, fundamental in the method of the PRISMA protocol, were not followed such as review protocol registration, detailed study selection criteria, data collection process, risk of bias within and across studies, and additional analyses. Million et al. (8) grouped “clinical” studies together (studies that had direct access to patients) and “observational big data” studies together (that may present conflicts of interest and show no effect of HCQ) instead of doing meta-regressions based on study designs (i.e., RCT vs. observational study). Fiolet et al. (26) excluded several studies because of critical risk of bias (i.e., lack of statistical information and the assignment of treatment, unknown timing between measures and confounders) with HCQ and AZ combination therapy (47–49).

Outcome selection is concerning, as Million et al. (8) reported positive effects on the duration of symptoms such as cough, fever, and clinical care with analysis of 1 to 7 small sample size studies (50). In Fiolet's et al. meta-analysis (26), the type of estimate used for effect sizes was inconsistent and not clearly reported. They did not make a distinction between Risk Ratios (RR), which are usually used in cohort studies, and Hazard Ratios (HR) and Odds Ratios (OR), which are used in case-control studies. This can influence the analysis because OR tend to overestimate effects compared to RR when the selected outcome occurs frequently (51). In both meta-analyses, the selection of included studies, the degree of heterogeneity in these analyses, and the calculations of effect sizes make the veracity of the estimates uncertain. Many of the meta-analyses published had low statistical power, untreated heterogeneity and none used tools to evaluate potential risks of *p*-hacking (13, 27–46, 52).

## Discussion

### A Proposal for Conducting Meta-Analyses in Clinical Research

As discussed above, one potential bias in meta-analyses is “selection bias,” which may lead to inaccurate estimation of effect sizes. An important question is whether to incorporate unpublished pre-print studies, especially when the field has limited studies and there is urgency for reliable data. An argument against this approach could be that unpublished studies might not be as rigorous as published studies. From this point of view, unpublished studies should not be included in meta-analyses because the inclusion of poorly conducted research also introduces bias. However, having access to published and unpublished studies helps decide which studies to include in a meta-analysis based on a priori inclusion criteria [through pre-registered meta-analyses for instance (1), see Table 2].

Readers typically focus on the forest plot, which depicts the quantitative effects and level of uncertainty for each study included in the meta-analysis. Forest plots are great tools to visually assess heterogeneity (coupled with quantitative index such as *I*^2^ or *Q*-test) and pooled results (53). However, forest plots do not address publication bias and thus can mislead readers's conclusion if not presented with additional information such as a funnel plot.

One of the most widely used tools to assess publication bias is plotting the effect sizes for each study against an indicator for the precision to which each study estimated the effect size. In funnel-plots, studies will be plotted near the average effect size, while studies with low precision (e.g., small sample) will have effect estimates distributed on either side of the average effect, creating a funnel-shaped plot.

Although funnel plots are a widely used and reliable way to evaluate publication bias, another useful tool is the *p*-curve (5). The *p*-curve plots a proportion of observed *p*-values for each value of *p* in a set of studies. Because true effects are more likely to have smaller values of *p* (e.g., “*p* < 0.01”) than values around the arbitrary significant threshold of *p* < 0.05, a flat *p*-curve or a *p*-curve indicating a higher proportion of *p*-values between 0.04 and 0.05 is more likely to be an indicator of questionable research practices, sometimes referred to as *p*-hacking. Van Assen et al. (4) propose the use of another tool, *p*-uniform, to estimate population effect size in the presence of small to moderate heterogeneity (*I*^2^ < 50%).

Conducting sub-group analyses for observational, quasi-experimental, and experimental studies will also help evaluate the risk of bias of each study design (6). In cases of substantial heterogeneity, researchers can generate a homogenous group of studies based on theoretical or methodological criteria and then use the *p*-curve and *p*-uniform to estimate the average population effect sizes for each subgroup analysis (4). Additional tools can be useful to determine publication bias. For instance, selection models can adjust for suspected selective publication; Rosenthal's fail-safe *N* is used to estimate the number of unpublished studies necessary to overturn the significant results and Copas sensitivity approach uses regression models to evaluate publication bias (54).

The fact that statistically significant results are more likely to be published than non-significant results is a major source of publication bias. Additional sources of potential bias that should be addressed when possible include pipeline bias (non-significant results take longer to publish than significant results), subjective reporting bias (selective reporting of the results), duplicate reporting bias (results published in multiple sources), and language bias (non-native English speakers tend to publish non-significant findings in their native tongue) (54).

## Conclusion

Tensions over the use of HCQ for COVID-19 patients have unfortunately led some authors to disregard basic meta-analytical protocols. Concern over the quality of studies included in meta-analyses has also emerged in a recent comparative psychological study between meta-analytical findings and registered replication studies. The authors found that meta-analytical effect sizes significantly differed from the replication effect sizes for 12 of the 15 meta-replication pairs, and meta-analytic effect sizes were almost three times larger than the replication effect sizes (15). These inconsistencies call for caution when running and interpreting meta-analyses of new clinical studies.

## Data Availability Statement

The original contributions presented in the study are included in the article/supplementary material, further inquiries can be directed to the corresponding author/s.

## Author Contributions

JB and OZ contributed to the original idea and wrote the original manuscript. JB, OZ, and CR analyzed the relevant literature and updated the manuscript. JB, OZ, CR, and AH were all contributor in writing the final manuscript. All authors read and approved the final manuscript.

## Conflict of Interest

The authors declare that the research was conducted in the absence of any commercial or financial relationships that could be construed as a potential conflict of interest.

## Publisher's Note

All claims expressed in this article are solely those of the authors and do not necessarily represent those of their affiliated organizations, or those of the publisher, the editors and the reviewers. Any product that may be evaluated in this article, or claim that may be made by its manufacturer, is not guaranteed or endorsed by the publisher.

## Abbreviations

AZ: Azithromycin
COVID-19: Coronavirus disease 2019
CQ: Chloroquine
EUA: Emergency-use authorization
FDA: Food and Drug Administration
HCQ: Hydroxychloroquine
HR: Hazard ratio
ICU: Intensive care unit
IPD: Individual patient data
OR: Odd ratio
PRISMA: Preferred reporting items for systematic reviews and meta-analyses
RCT: Randomized controlled trial
RR: Risk ratio.
